# Derivation and application of an analytical rock displacement solution on rectangular cavern wall using the inverse mapping method

**DOI:** 10.1371/journal.pone.0188336

**Published:** 2017-11-20

**Authors:** Mingzhong Gao, Bin Yu, Zhiqiang Qiu, Xiangang Yin, Shengwei Li, Qiang Liu

**Affiliations:** 1 State Key Laboratory of Hydraulics and Mountain River Engineering, College of Water Resource & Hydropower, Sichuan University, Chengdu, China; 2 Key Lab. of Energy Eng. Safety and Mechanics on Disaster (Sichuan Univ), Ministry of Education, Chengdu, China; 3 Datong Coal Mine Group Co. Ltd., Datong, China; 4 Changsha Institute of Mining Research Co.,Ltd, Changsha, China; China University of Mining and Technology, CHINA

## Abstract

Rectangular caverns are increasingly used in underground engineering projects, the failure mechanism of rectangular cavern wall rock is significantly different as a result of the cross-sectional shape and variations in wall stress distributions. However, the conventional computational method always results in a long-winded computational process and multiple displacement solutions of internal rectangular wall rock. This paper uses a Laurent series complex method to obtain a mapping function expression based on complex variable function theory and conformal transformation. This method is combined with the Schwarz-Christoffel method to calculate the mapping function coefficient and to determine the rectangular cavern wall rock deformation. With regard to the inverse mapping concept, the mapping relation between the polar coordinate system within plane *ς* and a corresponding unique plane coordinate point inside the cavern wall rock is discussed. The disadvantage of multiple solutions when mapping from the plane to the polar coordinate system is addressed. This theoretical formula is used to calculate wall rock boundary deformation and displacement field nephograms inside the wall rock for a given cavern height and width. A comparison with ANSYS numerical software results suggests that the theoretical solution and numerical solution exhibit identical trends, thereby demonstrating the method’s validity. This method greatly improves the computing accuracy and reduces the difficulty in solving for cavern boundary and internal wall rock displacements. The proposed method provides a theoretical guide for controlling cavern wall rock deformation failure.

## Introduction

In practical underground construction projects, owing to the benefits of relatively simple construction processes, rapid cavern formation, quickly and easily shaped cavern supports, and the high rate of cross-section utilization, rectangular cross-sectional caverns are increasingly employed in industrial and residential construction, transportation, mining, water resources and hydroelectric power generation, as well as defense projects. For instance, the largest underground shopping mall, Toronto’s PATH underground complex, occupies 370,000 m^2^ of space, and its foundation excavation has a full cross-sectional rectangular shape. Other examples include underground factories, urban subway stations, mining tunnels, underground hydroelectric powerhouses, underground energy storage warehouses, and underground shelters. Compared with tunnels of other cross-sectional shapes, the failure mechanism of rectangular cavern wall rock is significantly different as a result of the cross-sectional shape and variations in wall stress distributions [1]. The stability analysis of circular and elliptically shaped cavern wall rocks is informed by mature theoretical analysis methods [2–7]. However, an understanding of rectangular cavern wall rock deformation and damage characteristics is still at an initial exploration stage, and a mature theory has not yet been developed. Thus, a systematic in-depth study of the rectangular wall rock deformation mechanism has extremely important theoretical value and practical significance.

Complex variable functions can transform the boundaries of complicated shapes into simple shapes. Then, the solution obtained with the simply shaped boundary can be transformed back to the complicated boundary to obtain a solution for the original problem. This method is effective for solving complicated boundary problems. Therefore, researchers frequently use complex variable function theory to study rectangular cavern wall rock damage characteristics and mechanisms. On this basis, corresponding analytical equations used in wall rock deformation theory have been obtained. Muskhelishvili [8] applied a complex variable function to study the elastic mechanics of a planar problem. The result contributes to develop the computational theory of complex boundaries cavern. He et al. [9] treated a rectangular cavern as an equivalent circular cavern to facilitate a solution based on the equivalent Laurent series radius. But the approach is at the cost of computational accuracy. Savin et al. [10] used a complex variable function to study boundary stress distributions for caverns composed of isotropic or anisotropic materials other than boundary deformations. Sharma et al. [11] employed a complex variable function to analyze the stress concentration around circular, oval, round, triangular and quadrilateral caverns in an infinite plane. They suggest that the loading angle and corner radius are the key factors affecting the value of stress concentration. Huo et al. [12] used complex variable theory and conformal mapping to develop a solution for deep rectangular structure with a far-field shear stress. The solution showed that structural deformations depend on the relative stiffness between the structure and the surrounding ground, and on the shape of the structure. Charles et al. [13] presented a simple approximate solution for an artificial rectangular hole using complex variable theory and introducing a correction factor. Lv et al. [14] employed an optimization technique to obtain a mapping function for caverns with an arbitrary sectional shape. Akbarov et al. [15] used the framework of the three-dimensional theory of elasticity under a plane-strain state to study the influence of the initial stretching of a simply supported plate strip containing a rectangular hole. Grigorios et al. [16] made use of dynamic centrifuge tests and ABAQUS numerical modeling to research the seismic behavior of rectangular tunnels in soft soils; this study constitutes an important step in the development of appropriate specifications for the seismic design of rectangular shallow tunnels.

The deformation characteristics of a rectangular cavern have been described in different directions and levels by the researchers above. This research has had a significant guiding influence on subsequent in-depth discussions of rectangular cross-sectional cavern wall rock stability. However, most studies have been based on the transformation of a rectangular planar area into an area with a simple boundary shape, such as inside or outside a unit circle. This approach is especially suitable for analyzing typical points on the side of a hole (e.g., two sides, a top arch, and floor heave). However, rectangular plane Cartesian coordinates transform to multiple power functions of a unit circle in polar coordinates, so when this approach is used to solve for the displacement field at multiple points, the corresponding transformation has multiple solutions that are identified while a rectangular body is being mapped to corresponding unit circles. To obtain the corresponding unit circle coordinates, a determination based on relevant conditions is needed, thus causing the computation process to become extremely complex. However, according to the inverse mapping method [17], a one-to-one relationship exists where unit circle coordinate points are mapped to rectangular coordinates. Thus, this method greatly reduces the difficulty in solving for cavern boundary and internal wall rock displacements. This paper theoretically analyzes deformation at a rectangular cavern boundary and inside the wall rock, which enables the derivation of a corresponding function based on inverse mapping. The horizontal and vertical deformation displacements can be obtained for any point on the rectangular boundary and inside the wall rock. The results are compared with results calculated using ANSYS numerical software to verify the reliability of the proposed method. Therefore, the use of the inverse mapping method can greatly improve efficiency when solving for rectangular cavern internal wall rock displacements.

## Rectangular cavern displacement field solutions based on the inverse mapping method

### Complex variable function solutions for conventional rectangular cavern displacement

**Fig 1:**
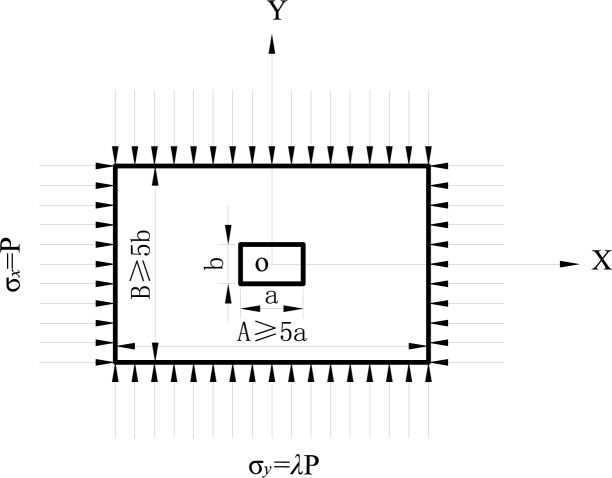
Mechanical model for rectangular cavern wall rock.

To study rectangular cavern deformation characteristics, a mechanical model has been constructed, as shown in Fig 1. This type of problem is treated as a hole-opening strain problem in an infinite plane because the cavern length exceeds the section dimensions. P represents the overlying strata pressure on the model, while λP represents the surrounding rock’s pressure on the model, where λ is the lateral pressure coefficient. The cavern dimensions are set to a×b, and the model dimensions are set to A×B. According to St. Venant’s principle [18], the stress and strain corresponding to the opening of the cavern act only within a radius of three times the distance from the center of the cavern. Thus, to eliminate this boundary effect, this study uses a mechanical model that is five times the size of the cavern.

Conformal transformation is applied, and the cavern is mapped onto the unit circle in the *ς* plane based on complex variable theory [19]. Conformal transformation is a method that is used to transform the area outside a hole with a specified shape onto the region either outside or inside a unit circle The key to this method is assessing how the conversion analytical function should be determined. A mapping function Z = w(ς) transforms the rectangle into a unit circle in the complex plane to facilitate study. Cavern wall rock deformation is determined by two analytical functions, φ(ς) and ψ(ς), based on elastic mechanics theory [20], as given by:

$$2G(u+iv)=k\varphi (\varsigma )-\frac{\omega (\varsigma )}{\omega^{\prime }(\varsigma )}\bar{\varphi^{\prime }(\varsigma )}-\bar{\psi (\varsigma )}$$ (1)

In the equation, *k* = 3–4*μ*, G is the shear modulus of the rock such that $G=\frac{E}{2(1+\mu )}$, *E* is the rock lithology modulus, and *μ* is Poisson’s ratio. Assuming that the polar coordinates of any point in the *ς* plane are (ρ, θ), *ς* = *ρ*e^*iθ*^ = *ρ*(cos*θ* + *i*sin*θ*). Eq (1) shows that only three complex variable analytical functions, ω(*ς*), φ(*ς*) and ψ(*ς*), need to be solved. The real and imaginary parts are separated to obtain displacement components u and v for any point. A mapping function must be calculated via conformal transformation for caverns with arbitrary sectional shapes.

### Mapping function solution based on the Schwarz-Christoffel method

The Z plane is mapped to a unit circle in the *ς* plane based on the mapping method provided by Schwarz-Christoffel. The general form of conformal transformation is:

$$\omega =K\int \left[{\left(z-x_{1}\right)}^{\frac{a_{1}}{\pi }-1}{\left(z-x_{2}\right)}^{\frac{a_{2}}{\pi }-1}\ldots {\left(z-x_{k}\right)}^{\frac{a_{k}}{\pi }-1}\right]dz+c$$ (2)

where α_1_, α_2_… α_k_ refer to the polygon mapping angles and *x*_1_, *x*_2_… *x*_k_ refer to the mapping positions. Based on complex variable function theory, *n* = 4 in the equation and *c* = 0 at the boundary when the Z plane represents an elastic body with a rectangular external boundary. Therefore, the rectangular equation converts to:

$$Z=\omega (\varsigma )=R\int_{0}^{\varsigma }\left[{\left(t-a\right)}^{\frac{\beta_{1}}{\pi }-1}{\left(t-b\right)}^{\frac{\beta_{2}}{\pi }-1}{\left(t-c\right)}^{\frac{\beta_{3}}{\pi }-1}{\left(t-d\right)}^{\frac{\beta_{4}}{\pi }-1}\right]dt$$ (3)

where *β*_*1*_, *β*_*2*_, *β*_*3*_, and *β*_*4*_ represent the four vertices of the rectangle and the complex variable transformations of *a*, *b*, *c*, and *d* are:

$$\begin{array}{l} a=e^{i(\frac{\pi }{2}-k\pi )},\\ b=e^{i(-\frac{\pi }{2}+k\pi )},\\ c=e^{i(\frac{\pi }{2}+k\pi )},\\ d=e^{i(\pi +k\pi )} \end{array}$$ (4)

The previous expressions are substituted into Eq (4), and *q*(*t*) is used to represent the integrand:

$$q(t)=\sqrt{\left(1+2t^{2}\;\cos2k\pi +t^{4}\right)}.$$ (5)

*q*(*t*) is expanded in the neighborhood of the zero point and substituted to obtain:

$$Z=x+iy=\omega (\varsigma )=R(\varsigma +c_{0}+\frac{c_{1}}{\varsigma }+\frac{c_{2}}{\varsigma^{2}}+\ldots +\frac{c_{n}}{\varsigma^{n}}\ldots )$$ (6)

where *c*_*n*_ is the mapping function coefficient, the value of which is $c_{n}=\frac{1}{n!}q^{\left(n\right)}(t){|}_{t=0}$ (the same below), and *R* is a constant that reflects the size of the rectangular cavern.

For the rectangular shape analyzed in this paper and according to the Schwarz-Christoffel equation, it is found that *c*_0_ = 0, *c*_1_ = cos2*kπ*, *c*_2_ = 0, *c*_3_ = -sin^2^2*kπ*/6, *c*_4_ = 0, *c*_5_ = -sin^2^2*kπ*cos2*kπ*/10, c_7_ = (10cos8*kπ*-8cos4*kπ*-2)/896. Thus, we obtain:

$$\left\{ \begin{array}{l} x=R(\rho \cos\theta +\frac{\cos2k\pi \cos\theta }{\rho }-\frac{\sin^{2}2k\pi }{6\rho^{3}}\cos3\theta +\ldots )\\ y=R(\rho \sin\theta -\frac{\cos2k\pi \sin\theta }{\rho }+\frac{\sin^{2}2k\pi }{6\rho^{3}}\sin3\theta +\ldots ) \end{array}\right..$$ (7)

When *ρ* = 1, θ = 0 and θ = π/2, based on Eq (7), *k* and *R* can be attained when *a* and *b* are given. Eq (7) shows that polar coordinates have multiple solutions when the Z plane coordinates (*x*, *y*) are given, and the corresponding polar coordinates should be determined based on the relevant condition. Only one corresponding Z plane coordinate exists when polar coordinates are given. The transformation from the *ς* plane unit circle outer domain to the original plane hole outer domain is called inverse mapping.

### Rectangular cavern wall rock displacement field calculation based on the inverse mapping method

The infinity neighborhood expressions for φ(*ς*) and ψ(*ς*) can be written as Eqs (8) and (9) when points at infinity possess bounded stress,

$$\varphi (\varsigma )=-\frac{1}{2\pi (1+\kappa )}(X+iY)\ln\varsigma +(B+iC)\varsigma +\varphi_{0}(\varsigma ),$$ (8)

$$\psi (\varsigma )=\frac{1}{2\pi (1+\kappa )}(X-iY)\ln\varsigma +(B^{\prime }+iC^{\prime })\varsigma +\psi_{0}(\varsigma ).$$ (9)

The excavation boundary force is a balanced force system (X = Y = 0), yielding:

$$\varphi (\varsigma )=(B+iC)\varsigma +\varphi_{0}(\varsigma ),$$ (10)

$$\psi (\varsigma )=(B^{\prime }+iC^{\prime })\varsigma +\psi_{0}(\varsigma ).$$ (11)

The Cauchy integral gives:

$$\varphi_{0}(\varsigma )+\frac{1}{2\pi i}\underset{\sigma }{\int }\frac{\omega (\sigma )}{\bar{\omega^{\prime }(\sigma )}}\frac{\bar{\varphi_{0}{}^{\prime }(\varsigma )}}{\sigma -\varsigma }d\sigma =\frac{1}{2\pi i}\underset{\sigma }{\int }\frac{f_{0}}{\sigma -\varsigma }d\sigma ,$$ (12)

$$\psi_{0}(\varsigma )+\frac{1}{2\pi i}\underset{\sigma }{\int }\frac{\bar{\omega (\sigma )}}{\omega^{\prime }(\sigma )}\frac{\varphi_{0}{}^{\prime }(\varsigma )}{\sigma -\varsigma }d\sigma =\frac{1}{2\pi i}\underset{\sigma }{\int }\frac{\bar{f_{0}}}{\sigma -\varsigma }d\sigma .$$ (13)

By solving Eq (12) to obtain $\varphi_{0}(\varsigma )=\overset{n}{\underset{k=1}{\sum }}c_{k}\varsigma^{-k}$ and substituting this into Eq (13), we obtain *ψ*_*0*_(*ς*). Then by substituting *φ*_*0*_(*ς*) and *ψ*_*0*_(*ς*) into Eqs (10) and (11), *φ* (*ς*) and *ψ* (*ς*) can be derived. Further, displacements *u* and *v* in the X and Y directions can be obtained:

$$\begin{array}{l} 2G(u+iv)=\kappa \overset{n}{\underset{\kappa =1}{\sum }}a_{\kappa }\varsigma^{-k}+\frac{\overset{n}{\underset{k=1}{\sum }}ka_{k}\rho^{-2(k+1)}\varsigma^{k+1}}{1-\overset{n}{\underset{k=1}{\sum }}kc_{k}\rho^{-2(k+1)}\varsigma^{k+1}}[\varsigma -\frac{\varsigma }{\rho^{2}}+\overset{n}{\underset{k=1}{\sum }}c_{k}\varsigma^{-k}(1-\rho^{2k})]\\ -\overset{n-2}{\underset{k=1}{\sum }}S_{k}\rho^{2k}\varsigma^{-k}-\frac{PR}{2}(1-\lambda )\overset{n}{\underset{k=1}{\sum }}c_{k}\rho^{2k}\varsigma^{-k}+\frac{PR}{2}(1+\lambda )\frac{\varsigma }{\rho^{2}} \end{array}$$ (14)

λ represents the lateral pressure coefficient. Variables a_k_ and s_k_ represent the relevant calculation coefficients for the mapping coefficient c_k_; they can be calculated using the Laurent series (Saint-Venant. 1855). When a point (ρ, θ) is given, based on the mapping function (7), the coordinates of this point can be calculated in the Z plane.

In the same way, the shear stress at the hole side is obtained when the boundary of the rectangular cavern is not loaded:

$$\sigma_{\theta }=P\left(1+\lambda \right)+\frac{4}{R}\cdot \frac{AC+BD}{A^{2}+B^{2}}$$ (15)

where: $A=-\cos(n+1)\theta +\overset{n}{\underset{k=1}{\sum }}kC_{k}\cos(n-k)\theta$, $B=-\sin(n+1)\theta +\overset{n-1}{\underset{k=1}{\sum }}kC_{k}\sin(n-k)\theta$, $C=\overset{n}{\underset{k=1}{\sum }}ka_{k}\cos(n-k)\theta$, $D=\overset{n-1}{\underset{k=1}{\sum }}ka_{k}\sin(n-k)\theta .$

## Case study

**Fig 2:**
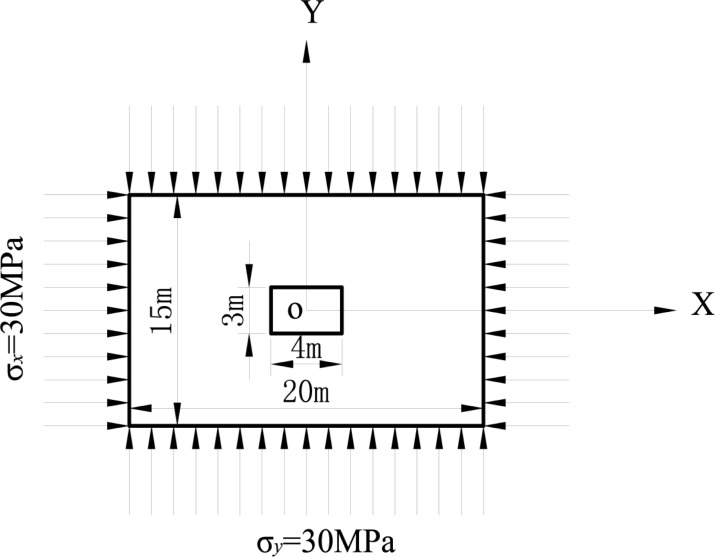
Rectangular cavern mechanical model example.

**Fig 3:**
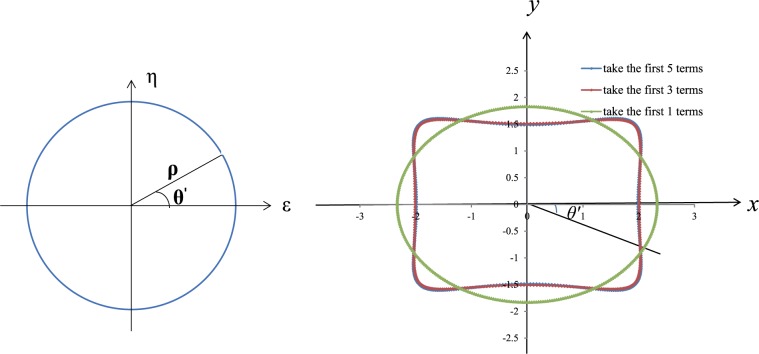
Mapping between the *ς* plane unit circle and Z plane rectangle.

Fig 2 illustrates a deep underground rectangular cavern with a width of 4 m, a height of 3 m and an initial ground stress given by (σ_x_ = 30 MPa, σ_y_ = 30 MPa, σ_z_ = 0). The wall rock’s basic mechanical parameters are listed in Table 1. Based on the mapping function (7), we can obtain: k = 0.23 and R = 2.0805, then use the Schwarz-Christoffel formula to get c_1_ = 0.125, c_2_ = 0, c_3_ = **-**0.164, c_5_ = **-**0.012, and c_7_ = 0.016. The corresponding coordinates of the intersection angle in the unit circle’s circumference can be calculated via inverse mapping. The corresponding intersection angle θ′can then be calculated in the rectangular plane. When taking different negative power terms, the mapping between the unit circle and rectangle can be shown in Fig 3. The figure shows that when we take the expansion terms to 1, the mapping tends to an ellipse, with the expansion terms increased to 3, we can find that the mapping tends to rectangular geometries where the corner is rounded rather than at a right angle shape. When we add the terms to 5, it can be seen that when n≥5 (where n represents the number of expansion terms), the coefficient of mapping functions are small enough that the impact on the result of mapping can be ignored. Therefore, we set the negative power terms to n = 3. From the Laurent series, a_1_ = p_r_(1+λ) c_1_/[2(c_3_-1)], a_2_ = -p_r_(1+λ) c_2_/2, a_3_ = -p_r_(1+λ) c_3_/2 and S_1_ = a_1_×c_3_. MATLAB software is used to translate the calculation formula (14), execute the transformation between the cavern dimension related polar coordinates and Cartesian coordinate system and to store the mapping parameter calculation procedure so that the displacement can be calculated at any specific position on the cavern boundary or inside wall rock. The required parameters are calculated as k = 0.23, r = 2.0805, c_1_ = 0.125, c_2_ = 0, c_3_ = -0.164, a_1_ = -5.7122 ×106, a_2_ = 0, a_3_ = 8.7032 ×106 and S_1_ = 0.937 ×106.

**Table 1:** Cavern wall rock mechanical parameters.

Elastic modulus E/GPa	Poisson ratio *μ*	Tensile strength *σ*_*c*_/MPa	Compressive strength *σ*_*t*_/MPa
25	0.15	30	3

**Fig 4:**
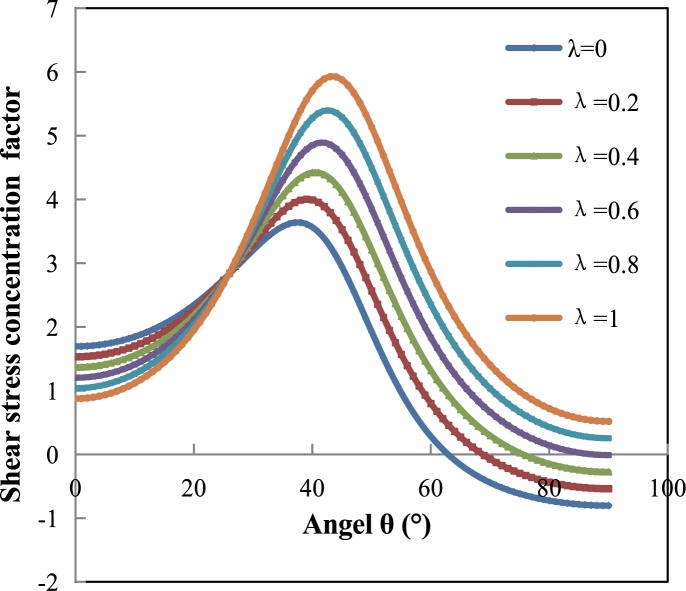
The curves of shear stress concentration for cavern boundary displacement.

According to the above theoretical results, λ will affect it as a variable in the solution formula of *σ*_*θ*_. Combining the different boundary angles of the cavity in Z plane, the shear stress coefficient under diverse boundary angle distribution with different lateral pressure coefficient can be obtained. As the model is symmetrical, this paper takes the 1/4 structure to analysis, the calculation results are shown in Fig 4. We can see that the variation trends of shear stress coefficient under different side pressure coefficients are consistent and all of these showed an initial increase followed by a decrease, and the maximum stress concentration coefficient occurs at an angle of 35° to 40° to the horizontal, i.e., near the right angle of a rectangle. In addition, when the value of λ is relatively small, tensile stress will appear surrounding the cavern, and with the increase of λ, the tensile stress decreases gradually until compressive stress appears. When λ≥0.8 the stress surrounding the cavern is all compressive stress.

**Fig 5:**
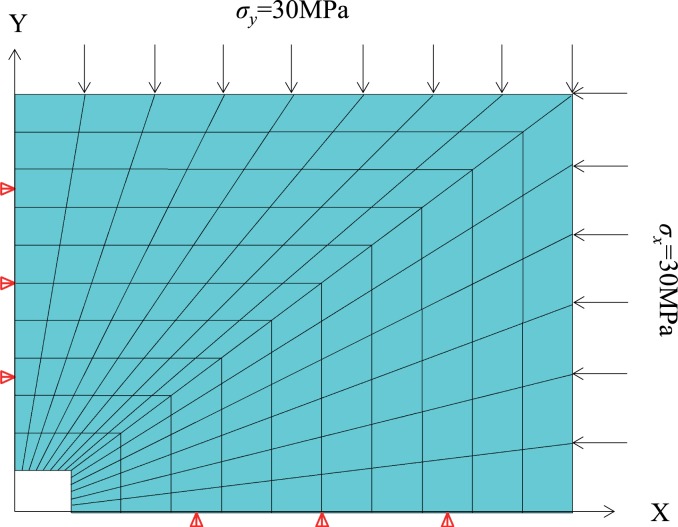
The chart of ANSYS numerical model.

**Fig 6:**
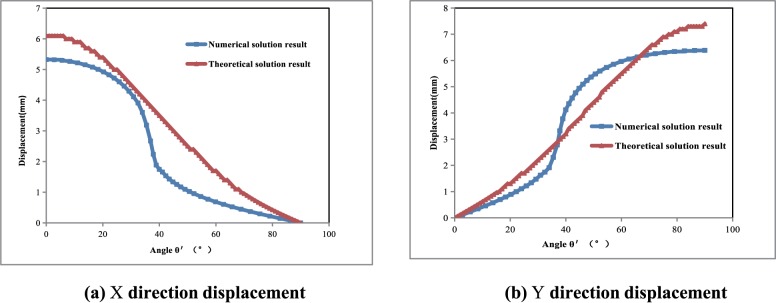
Comparison of theoretical solution versus numerical solution for cavern boundary displacement.

The X direction displacement around the cavern gradually decreases from 6.1mm (in the middle of the lateral wall) to 0 mm (roof) with increasing angle. The Y direction displacement displays an opposite trend, gradually increasing from 0 mm (in the middle of the lateral wall) to 7.4 mm (at the roof) with increasing angle. To verify the accuracy of the theoretical calculation, ANSYS finite element analysis software is employed to simulate wall rock boundary deformation under stress. The numerical calculation model is shown in Fig 5; the size of the cavern is 2×1.5m and the model size is 11 times that of the cavern, i.e., a horizontal length of 22m, a vertical height of 16.5m, with a total mesh of 140. Additionally, the elastic model is used for numerical simulations. The upper and right lateral surfaces are stress boundaries, with the stress given by σ_x_ = σ_y_ = 30MPa. A displacement boundary condition is applied to the floor and left lateral surfaces. The boundary displacement calculation results are shown in Fig 5 in the blue curve. The maximum horizontal direction displacement is 5.3 mm, and the maximum vertical direction displacement is 6.4 mm. Comparative analysis indicates that the maximum displacement errors between the theoretical and numerical simulation calculation results are 14.5% in the horizontal direction and 15.6% in the vertical direction (as in Fig 6). Moreover, the maximum displacement errors in both directions occur at the vertices of the rectangular cavern. A possible source of the above errors is the process of mapping the unit circle to a rectangle (as in Fig 3), in which only the first three terms of the inverse mapping function are used. In doing so, the four vertices became four circles of small radii, which is different from an actual rectangular shape. Therefore, errors arise between the theoretical and numerical simulation values, and the errors are mainly concentrated in the vertex area. The errors are relatively small and within the acceptable range. Therefore, both the analytical results and the comparison suggest that the proposed method can be used to calculate the cavern boundary elastic displacement.

**Fig 7:**
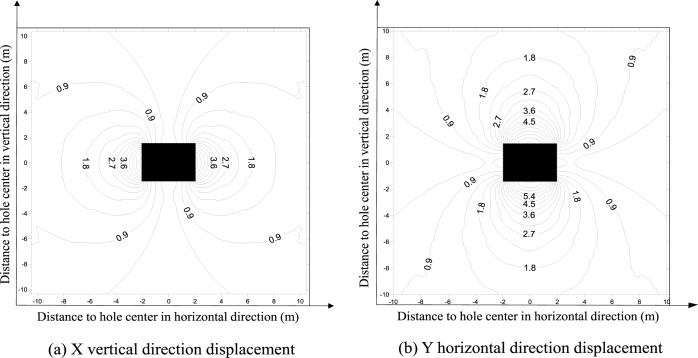
Theoretical cavern wall rock interior displacement solution.

The evolution pattern of displacement inside the wall rock following cavern excavations (ρ≠1 or ρ = 2, 3, 4, 5, 6, 7 and 8) is substituted into Eq (14) to calculate the wall rock interior displacement field nephogram, as shown in Fig 7. Both the vertical and horizontal direction displacements decrease when the distance between the monitoring point and hole opening increases. This variation pattern also matches the actual scenario, which further validates the theory.

Basically, the method for calculating the displacement of surrounding rock of cavern is put forward tentatively, which provides a new way for calculating the displacement of cavern. However, the shortcoming of the method is that the influence of complex geological conditions such as faults, joints and folds on the calculation results has not been considered yet. When it comes to the real engineering, an intensive research should be conducted.

## Conclusions

1. A mapping function coefficient has been calculated and theoretical expressions for X- and Y-direction displacements have been derived for polar coordinates, based on the semi-infinite elastic body assumption, complex variable elastic theory, the conventional Laurent series complex method and Schwarz-Christoffel mapping. The proposed calculation method can be used as a reference for calculating rectangular cavern boundary displacement and the area of influence due to excavation.
2. Inverse mapping is investigated between polar coordinates in the unit circle plane and Cartesian coordinates in the rectangular plane. The transformation from a polar coordinate θ to an intersection angle θ′ between Cartesian coordinates and the X axis is also investigated. A relationship diagram is established between θ′ and horizontal and vertical direction displacement, providing an intuitive representation of wall rock deformation at a specific cavern boundary position.
3. Detailed comparisons suggest that the boundary displacement deformation rule calculated using the proposed method matches the ANSYS numerical simulation results. Wall rock interior displacement field nephograms are presented to verify the relevant elastic displacement calculations for a rectangular cavern via the proposed method.
4. A cavern’s axial strain *ς*_z_ should be taken into account and cannot be treated as a planar strain problem if the studied cavern is relatively short. This type of problem must be solved using a complex variable function. Negative power terms in the mapping function should be accordingly increased to obtain a more accurate result.
